# Development and evaluation of a broadly reactive reverse transcription recombinase polymerase amplification assay for rapid detection of murine norovirus

**DOI:** 10.1186/s12917-018-1736-1

**Published:** 2018-12-14

**Authors:** Lei Ma, Fanwen Zeng, Feng Cong, Bihong Huang, Yujun Zhu, Miaoli Wu, Fengjiao Xu, Wen Yuan, Ren Huang, Pengju Guo

**Affiliations:** 1 Guangdong Key Laboratory of Laboratory Animals, Guangdong Laboratory Animals Monitoring Institute, Guangzhou, China; 2 College of Veterinary Medicine, South China Agricultural University, Guangzhou, 510640 China

**Keywords:** Murine norovirus, Recombinase polymerase amplification, Genetic diversity, Detection

## Abstract

**Background:**

Murine norovirus (MNV) is recognized as the most prevalent viral pathogen in captive mouse colonies. The rapid detection assay for MNV would be a useful tool for monitoring and preventing MNV infection. A recombinase polymerase amplification (RPA) assay was established in this study to provide a solution for rapid and sensitive detection of MNV.

**Results:**

The detection limit of the RT-RPA assay for the detection of MNV was 1 × 10^2^ copies of RNA molecules per reaction. The assay was specific since there was no cross-reaction with other common murine viruses. In addition, the broad reactivity of the RT-RPA assay was validated using the synthesized template carrying seven point mutations among several MNV strains. The MNV RT-RPA assay could detect as few as 1 × 10^2^ copies of the mutant per reaction, suggesting the assay could be broadly reactive against a large diversity of MNV strains. Forty eight clinical samples including 16 gastric tissue specimens, 16 cecal tissue specimens and 16 fecal specimens were tested for the validation of the new developed RT-RPA assay. The detection results of RT-RPA and RT-qPCR for clinical samples were very similar, except that a gastric tissue sample which was positive by RT-qPCR, with a RNA titer of 27 copies, was negative by RT-RPA.

**Conclusions:**

A broadly reactive RT-RPA assay was successfully established for MNV detection.

## Background

Norovirus is a class of viral pathogens that could infect a variety of hosts [1]. Norovirus have been tentatively classified as five genogroups based on the sequences of major capsid protein (VP1) [1]. In a previous study, standard norovirus classification tree made with the alignment of 68 NoV sequences was proposed for nomenclature: 29 genetic clusters are classified in the 5 genogroups, 8 in GI, 17 in GII, 2 in GIII, and 1 each in GIV and GV [1]. GI, GII, and GIV strains, are found in humans, and GIII and GV strains are found in cows and mice, respectively. MNV, belongs to genogroup GIII, is one of the most common pathogens in laboratory mice [2, 3].

Since the prototype strain MNV-1 was firstly detected in immunocompromised mice [3], multiple strains of MNV have been discovered and/or isolated from the laboratory and wild rodents [4–6]. Although different MNV strains exhibited the considerable genetic and biological diversity, they belonged to a single serotype determined by cross-neutralization test [7]. Serological investigations in North America, Europe and Japan demonstrated the high prevalence of specific antibodies against MNV in laboratory rodents [8–10]. Currently, MNV has become the most prevalent pathogen in laboratory mice worldwide.

The mice are the most commonly used laboratory animals in biomedical research. The high prevalence of MNV in laboratory mice may interfere in the result of biomedical research. Several experiments have been performed to investigate the effects of MNV infection on scientific research. For examples, experimental infection of the mice revealed that MNV-1 lead to the persistent infection and long fecal virus shedding [2]. MNV-4 infection induced multiple inflammatory hallmarks of human Crohn’s disease in Atg16L1^HM^ mice after dextran sodium sulfate administration [11]. Whereas, another study reported that transient or persistent norovirus infection didn’t alter the pathology of intestinal inflammation and fibrosis induced by *Salmonella Typhimurium* infection in C57BL/6 mice [12]. As no clear conclusion can be made from the previous studies regarding to the effects of MNV infection on biomedical research in mice, the use of MNV-free mice was recommended for fundamental research and drug safety evaluation. Thus, the rapid and sensitive detection methods for MNV infection played a critical role in the quality control of the laboratory mice.

To monitor and detect MNV rapidly and specifically, several molecular methods such as reverse transcription polymerase chain reaction (RT-PCR) assays and RT-quantitative PCR (RT-qPCR) assays have been developed [8, 13–15]. Considering that the RT-PCR and RT-qPCR methods are time-consuming, the loop-mediated isothermal amplification (LAMP) method for detection of MNV was developed [16]. However four to six primers were required in the LAMP assay [16]. Recombinase polymerase amplification (RPA), another isothermal amplification method, which requires only two primers, has been recently used for pathogen detection and only takes less than 30 min to perform the amplification reaction [17–21]. RPA primers bind to DNA template by bacterial recombinase enzyme, then the extension and amplification reaction is initiated by an isothermal polymerase [22]. These enzymes are in concert with a reverse transcriptase and a fluorescent probe for real-time detection of the viruses containing RNA genomes [23].

Previous study has reported the RT-RPA assay for detection of human norovirus [24]. However, the whole genome sequence of human norovirus shared poor identity with MNV [2]. The RT-RPA assay used for human norovirus cannot be applied to the detection of MNV. Thus the aim of the present study is to establish a broadly reactive RT-RPA assay for rapid and sensitive detection of MNV which is widely present in laboratory mice across the world.

## Results

### Screening of the primer sets

Six primer sets were screened by the basic RT-RPA assay using the 10^4^ copies RNA standard as the template. As clearly showed in Fig. 2a, the product of the primer set f5/r5 emitted the strongest light under ultraviolet light. The probe was designed based on the target sequence of the primer set f5/r5. All the sequences of primers and probe used in this study were displayed in Table 1.

**Table 1:** Primers and probe

Name	Sequence	Amplicon length
MNV-F	ATGCATGGTGAAAAGTACTAT	788 bp
MNV-R	TAGAAAGAAGGCGACCAGAGA	
f1	CAGTCTTTGTGAATGAGGATGAGTGATG	189 bp
r1	AAAATTTTGGAAGATCCAGGGGTCAATTT	
f2	CAAATCAACCAAATTGACCCCTGGATCTT	151 bp
r2	ACCTCCATGTTCCCAACCCAGCCGGTGTACAT	
f3	CAAAATTTTGTCCAGTGCCCCCTTGGTGAGTT	135 bp
r3	AGGACCAGCTGAACCTCCATGTTCCCAACCCA	
f4	CACGCCACCGGTCTGTTCTGCGCTGGGTGC	154 bp
r4	AAGGAACAAGATCCTGGCCGCTGGCTTC	
f5	TTCCAAAATTTTGTCCAGTGCCCCCTTGGT	131 bp
r5	GCTGAACCTCCATGTTCCCAACCCAGCCGGTG	
f6	TTCAGCTGGTCCTCGCCGGCAATGCCTTTA	141 bp
r6	CCAGGGTGCGCACATCACACATGACATGTG	
probe	TCGAAACACCCCAGGCGAAATATTGTTTGA(BHQ)(THF)(FAM)GGCCCTCGGGCCAGG	

*BHQ1-dT* dT-fluorophore, *THF* tetrahydrofuran, *FAM-dT* dT-quencher group

### Sensitivity and specificity of the real-time RT-RPA

**Fig. 1:**
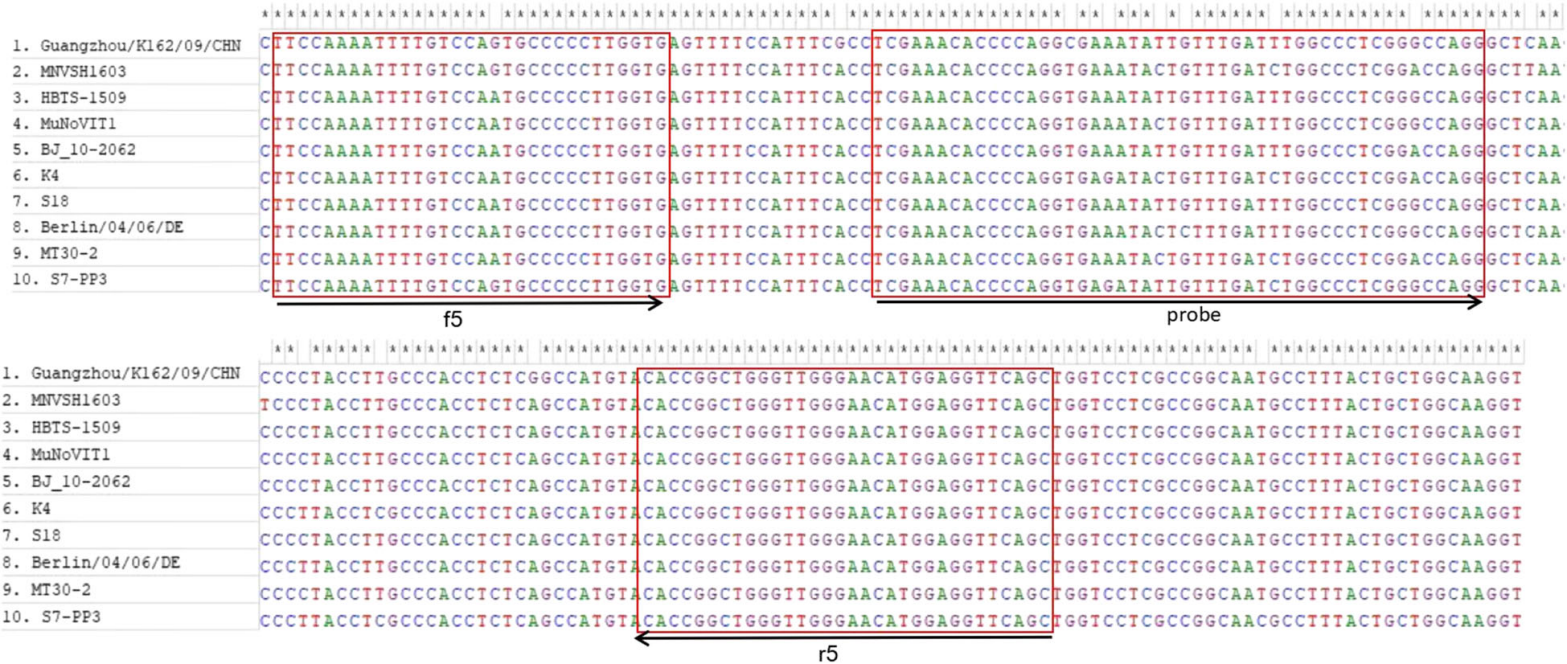
Alignment of nucleotide sequences from the ORF1–ORF2 junctions of 10 MNV strains. Nucleotide sequences of 10 MNV strains are aligned from nt 5220 to nt 5420 in Guangzhou/K162/09/CHN. Asterisks indicate consensus nucleotide sequences among the 10 MNV genes. The positions with no asterisk indicate bases that are different from the nucleotide sequence of Guangzhou/K162/09/CHN. The positions of RPA primers (forward primer: nt 5221–5250, reverse primer: nt 5352–5383) and probe (nt 5268–5315) are indicated in the red box

The detection limit of the RT-RPA assay was assessed using the 10-fold serially diluted RNA standards. As illustrated in Fig. 1b, the dynamic detection range of the assay ranged from 10^6^ to 10^2^ copies per reaction, demonstrating that the limit of detection of the assay was 10^2^ copies/μL molecular RNA. The RT-RPA assay was performed eight times on the serially diluted RNA standards, in which 8 out of 8 (8/8) runs were positive when viral load are 10^6^–10^2^ copies per reaction; 5/8 runs were positive when 10 copies per reaction; and 0/8 positive when 1 copy per reaction was used. To evaluate the reproducibility of the RT-RPA assay, semi-logarithmic regression analysis was performed using the data from the 8 runs (Fig. 3a). A probit regression analysis using the results of 8 runs was performed to determine the exact detection limit, and the results showed that RT-RPA could detect 70 RNA molecules in 95% of cases (Fig. 3b). The synthesized templates carrying seven point mutations were also tested by the RT-RPA assay to evaluate the broadly reactive capacity of the assay. The result showed that the RT-RPA assay can detect the mutant template as few as 10^2^ copies/μL molecular RNA (data not shown), indicating that the point mutations had no effect on the sensitivity of RT-RPA assay.

**Fig. 2:**
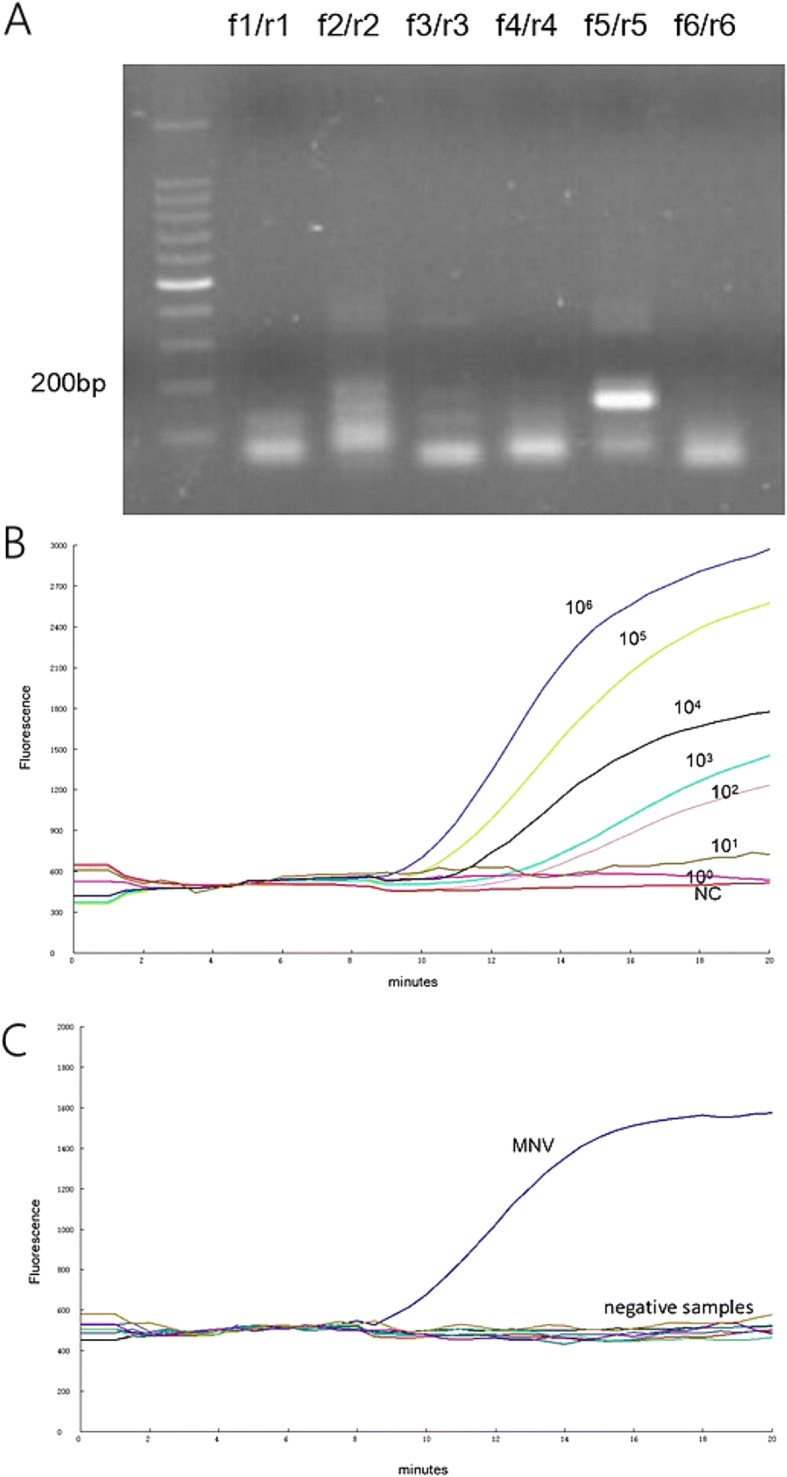
Primers sets screening; sensitivity and specificity of the RT-RPA assay. **a** The products of RT-RPA using six primers pairs were separated on agarose gel electrophoresis. **b** Fluorescence signal over time using the RNA standards ranging from 10^6^ to 10^0^ copies. **c** Specificity of the RT-RPA assay. TMEV, SeV, MHV, MPV, Reo-3 and distilled water were the negative samples

RNA genomes from other common murine viral pathogens were tested by the RT-RPA assay. Figure 2c revealed that the amplification curve was only observed for MNV; no amplification signal was detected for TMEV, SeV, MHV, PVM, Reo-3 and distilled water.

### Assay performance on clinical samples

**Fig. 3:**
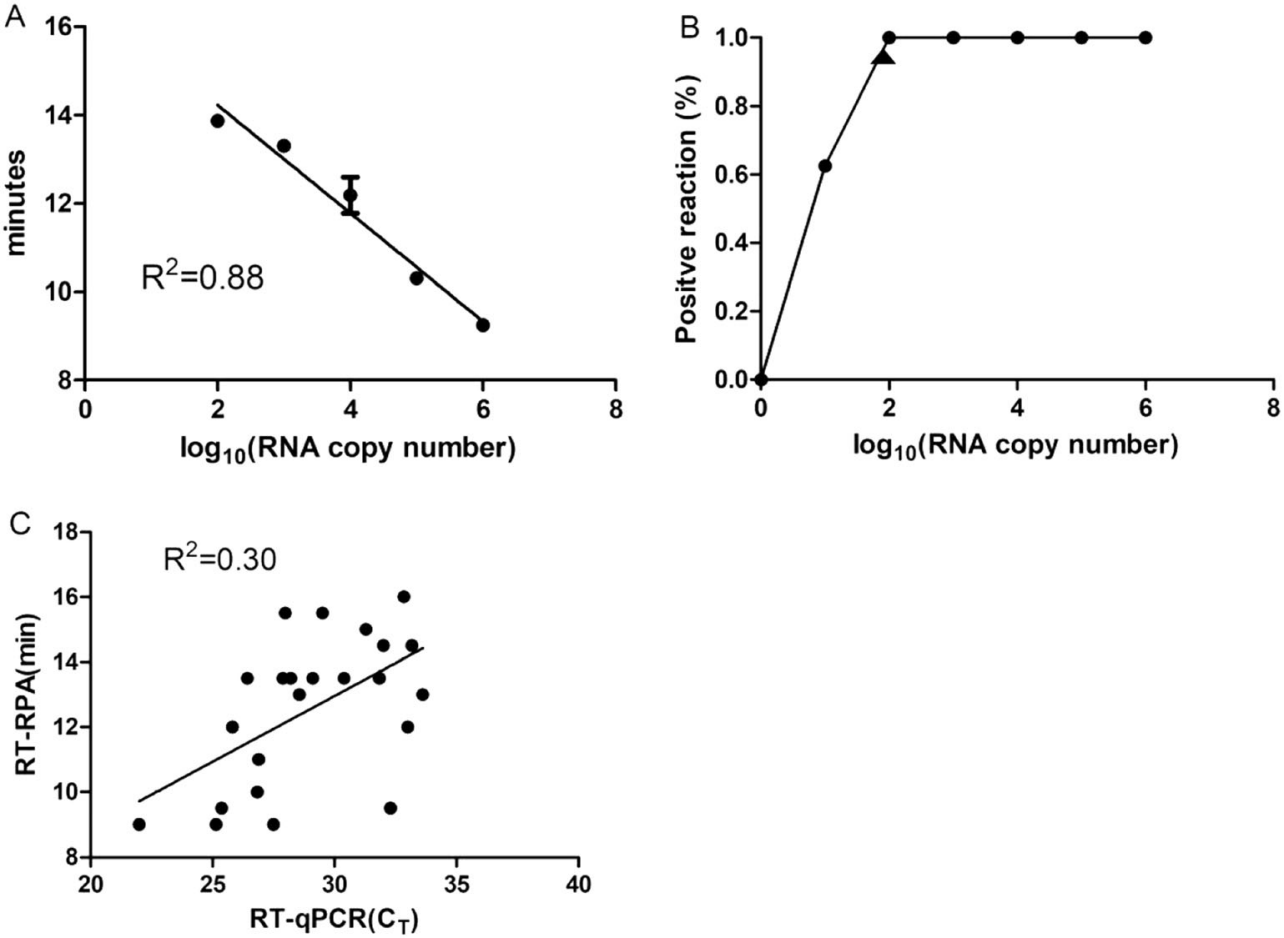
Performance of the RT-RPA assay. **a** Semi-logarithmic regression of the data collected from 8 runs using the RNA standard analyzed by GraphPad Prism 5.0. **b** Probit regression analysis using the data of the 8 runs. The detection limit at 95% probability (95 molecules) is depicted by a triangle. **c** Linear regression analysis of RT-RPA threshold time (TT, y axis) and RT-qPCR cycle threshold (CT) values (x axis) were performed by Prism software. R^2^ value was 0.3

The performance of RT-RPA was compared with probe-based RT-qPCR using RNA isolated from 48 murine samples. Eight of 16 cecal samples and 9 of 16 fecal samples were positive by both assays. In contrast, 6 of 16 gastric tissue samples were by RT-RPA while 7 of 16 gastric tissue samples were positive by RT-qPCR. The viral load in the gastric tissue sample which was negative by RT-RPA, was 27 copies per reaction determined by RT-qPCR, which was lower than the detection limit of the RT-RPA assay. Linear regression analysis demonstrated a poor correlation between C_T_ values determined by RT-qPCR and TT values determined by RT-RPA (Fig. 3c), suggesting the RT-RPA assay could not be applied to quantitative analysis of MNV viral loads in the samples. This phenomenon was also reported in the RT-RPA assay for detection of avian influenza virus in clinical samples [25].

## Discussion

In present study, a real-time RT-RPA assay was established for rapid and specific detection of MNV. The primer-probe set was designed based on the sequences of the highly conserved ORF1-ORF2 junction. This new assay could detect a broad range of genetically diverse MNV isolates with the same detection sensitivity.

MNV is considered the most prevalent viral pathogen in laboratory mice worldwide. MNV infection may affect the results of biomedical researches [16], so it is of vital importance to confirm the MNV-free status of the laboratory mice. Several real-time RT-PCR assays have been developed for detecting MNV. However, these techniques could not detect all the MNV strains due to the genetic diversity of MNV [5, 15, 26]. Thus two broadly reactive real-time RT-PCR assays were established to detect MNV infection [27, 28]. Although the real-time RT-PCR assay is sensitive and specific, it is expensive, time-consuming and labor-intensive. To overcome the shortcomings of the real-time RT-PCR, a broadly reactive LAMP assay was developed for detection of MNV. However, the LAMP assay required five primers, which increased the difficulty of primer design for MNV with genetic diversity. A portable and user-friendly method is therefore necessary for rapid detection of MNV on site. One promising solution is the RPA assay. Due to its convenience and portability, the RPA technique has been used for rapid detection of various pathogens, such as foot-and-mouth disease virus [29], Zika virus [30], human norovirus [24] and pathogenic bacteria [31]. Up to date, there is only one report about the RPA assay for the laboratory animal health monitoring [32].

Design of the primer set plays a vital role in the performance of the RPA assay. Up to now, no software is available for RPA primer design. The primers used in this study were manually designed following the manufacturer’s instruction. In this study, six primer sets were tested by the RPA assay, only one primer set (f5/r5) produced strong gene amplification signal. The probe was designed based on the target region of the primers set (f5/r5). The primer-probe set produced satisfied result (Fig. 2b). This result demonstrated that it is efficient and cost-effective to screen the primer sets through basic RPA assay, followed by gel electrophoresis.

Although the target region of the RT-RPA assay is highly conserved within the whole MNV genome sequence, it still possesses several point mutations among different strains that may affect the diagnostic sensitivity of the RPA assay. The sequences of 10 represent MNV strains in different countries were aligned. There are six different nucleotides at the binding site of the forward primer and the probe between the MNV strain Guangzhou/K162/09/CHN and strain K4. In addition, one more point mutation was present at other strains. To determine the effect of all the mutations on the performance of the RT-RPA assay, the templates containing all the seven point mutations were synthesized and tested by the RT-RPA assay. The result demonstrated that the mutations have no effect on the performance of the RT-RPA assay, which was consistent with previous studies proving that up to nine point mutations were tolerated [33, 34], suggesting that the RT-RPA assay could detect a diverse array of MNV strains.

The clinical performance of the RT-RPA assay was evaluated using 48 field samples. The MNV positive rate was 47.9% (23/48) determined by RT-RPA and 50.0% determined by RT-qPCR. The sensitivity of the RT-RPA technique for detection of MNV in clinical samples was 95.8% when compared with the RT-qPCR assay. Only one sample containing low RNA titer (27 RNA copies) was tested negative by RT-RPA. We believe that this was due to the low RNA copies (27 RNA copies) in the sample and low analytical sensitivity of the RT-RPA technique. However, the MNV RT-RPA assay was 10 times more sensitive than the RT-RPA for detection of human norovirus reported in a previous study [24]. It is worthy to note that the limit of detection of the human norovirus RT-RPA assay was performed on serially diluted RNA extract of stool. The sensitivity of present study was determined using a purified in vitro transcribed RNA rather than RNA extract of stool which may contain a lot of other RNA that interacts with recombinase proteins and thus can harm sensitivity. This may explained the difference of sensitivity between the two RPA assays. The RT-RPA assay could detect human norovirus in directly boiled stool, and displayed better resistance to inhibitors than a commonly used RT-qPCR assay [24]. It is promising to detect MNV in directly boiled animal tissue or stool samples by the RT-RPA assay in the future. This procedure would be valuable to reduce sample processing time and increase portability.

## Conclusion

A rapid, sensitive and specific method for detection of MNV was successfully established based on RPA assay. The RT-RPA had several advantages over RT-qPCR, including: (i.) a cost-effective portable device is sufficient; (ii) quicker time-to-result; (iii) the use of enzyme pellet in one tube reduces the laborious work and the likelihood of contaminant; (iv.) tolerance to common PCR/qPCR inhibitors [24]; (v.) the potential of point-of-care detection of the clinical samples. Thus, the assay provides an easy-to-use platform for rapid detection and monitoring of MNV infection which circulates in laboratory mice across the world.

## Methods

### Virus

MNV Guangzhou/K162/09/CHN strain was isolated and preserved in our laboratory [35], Theiler’s mouse encephalomyelitis virus (TMEV) BeAn8386 strain, sendai virus (SeV) Sendai/52 strain, mouse hepatitis virus A59 strain (MHV), reovirus type 3 (Reo-3) Dearing strain, pneumonia virus of mice (PVM) number 15 strain were purchased from American Type Culture Collection (ATCC). Nucleic acid for lymphocytic choriomeningitis virus (LCMV) Armstrong strain were kindly provided by Dr. Zheng Ming He, from the Laboratory Animal Institute, National Institutes for Food and Drug Control, China.

### Generation of MNV RNA standard

The ORF1-ORF2 junction sequence on MNV genome was demonstrated to be highly conserved [16]. A pair of primers (MNV-F: 5’-ATGCATGGTGAAAAGTACTAT-3′, MNV-R: 5’-TAGAAAGAAGGCGACCAGAGA3’) were designed and used to amplify the highly conserved ORF1-ORF2 junction. RNA of MNV strain Guangzhou/K162/09/CHN (Accession No: HQ317203) was used to amplify the target region by One Step RT-PCR kit (Takara, China). The reaction condition was set up as follows: reverse transcription (RT) at 50 °C for 30 min, denaturation at 95 °C for 5 min, 30 cycles of 94 °C for 30 s, 50 °C for 30 s and 72 °C for 45 s, a final extension step at 72 °C for 5 min. The amplicon was subjected to gel electrophoresis and recovered. The purified amplicon was cloned into the pGEM-T vector (Promega, USA) and designated as pGEM-MNV. The positive plasmid was confirmed by Sanger sequencing and in vitro transcribed using RiboMAX™ Large Scale RNA Production Systems (Promega, USA) according to the manufacturer’s instruction. The in vitro transcribed RNA was quantified by Nano2000 (GE, USA) and converted to copy number.

### Primer and probe design

There is no software available for RPA primer and probe design up to now. The primer sets were manually designed following the manufacturer recommendation (TwistDx), based on the sequence of MNV RNA molecular standard. Basic RPA assay was performed to screen the primer set with the best amplification efficiency. Thereafter, the probe was designed based on the target sequence of the primer pair.

### RT-RPA assay

Basic RT-RPA reaction was carried out in a 50 μl volume using the TwistAmp Basic RT kit (TwistDx, UK), which consisted of 30.1 μl of rehydration buffer, 1 μl of template, 2.1 μl of each primer (10 μM), 12.2 μl of water and 2.5 μl of magnesium acetate (280 mM). The microtubes were immediately placed in the heating block and incubated at 39 °C for 20 min. The RPA product was cleaned using a clean-up kit (Beyotime, China) to remove the inhibitors that might affect the agrose electrophoresis and separated on a 2% agarose gel.

Real time RT-RPA assay was performed using the TwistAmp exo RT kit. The reaction conditions for real-time RT-RPA were the same as for the basic RT-RPA, except that 0.6 μl of water was replaced by 0.6 μl of probe. The mircotubes were placed in the Deaou-308C tubescanner (DEAOU Biotechnology, China) set to 39 °C to start the reaction. After incubating for 4 min, the mircotubes were mixed, spun down, and placed back on the tubescanner for 16 min at 39 °C. A sample was considered positive when it generated an exponential amplification curve above the threshold of the negative control.

### Sensitivity and specificity of the RT-RPA assay

The transcribed RNA standard was 10-fold serially diluted by distilled water and used to evaluate the detection limit of the MNV real-time RT-RPA assay. Each RNA dilution was tested in 8 replicates. A semi-log regression was performed by plotting the threshold time against the log_10_ RNA copy numbers using Prism 5.0 software (GraphPad, USA). To determine the analytical sensitivity of the RT-RPA technique, a probit regression was performed using Prism 5.0 software (GraphPad, USA).

Due to the high genetic diversity of different MNV strains, the broad reactivity of the RT-RPA technique was investigated. Multiple alignments of sequences from 10 isolates retrieved from Genebank were performed. These 10 MNV strains were isolated from different countries and are the dominant strains in these countries, the point mutations at the primer-probe set may represent a large diversity of MNV. Six point mutations were present at the binding site of the probe and one mutation was present at the RPA forward primer (Fig. 1). The sequence containing all the seven point mutations were synthesized by Sangon Biotech (Sangon, China) and cloned in pGEM-T vector. The sensitivity of the RT-RPA assay using the mutants as the template was evaluated by the above method.

The specificity of the MNV RT-RPA assay was evaluated by testing a panel of viruses including TMEV, Reo-3, SeV, PVM and MHV.

### Detection of clinical samples

Forty eight frozen clinical samples (16 gastric tissue specimens, 16 cecal tissue specimens and 16 fecal specimens) from previous experiment stored at − 80 °C at our laboratory were subjected to the RT-RPA assay [36]. Homogenates of the tissues were produced with Dulbecco’s Modified Eagle Medium (DMEM). The homogenates after two freezing-thawing cycles were clarified by high-speed centrifugation and the supernatant fluids were collected for RNA extraction and RT-RPA assay. All the samples were also tested by the RT-qPCR method previously described [27]. Linear regression analysis of RT-RPA threshold time (TT) and RT-qPCR cycle threshold (C_t_) values were performed by Prism 5.0 software (GraphPad, USA).

## Abbreviations

MNV: Murine norovirus
RT-PCR: Reverse transcriptase polymerase chain reaction
RT-RPA: Reverse transcriptase recombinase polymerase amplification
